# One cancer, two stories: divergent estimates of gastric cancer burden in Korea

**DOI:** 10.1186/s13690-025-01801-2

**Published:** 2026-01-14

**Authors:** Chul Hyun, Yun Seo Kim, Sarah Soyeon Oh, Sung Hwi Hong, Jae Il Shin

**Affiliations:** 1 Section of Digestive Diseases, Yale School of Medicine, New Haven, CT United States; 2 Yonsei University College of Medicine, Seoul, Republic of Korea; 3 Institute for Global Engagement & Empowerment, Yonsei University, Seoul, Republic of Korea; 4 Severance Underwood Meta-Research Center, Institute of Convergence Science, Yonsei University, Seoul, Republic of Korea; 5 Department of Pediatrics, Yonsei University College of Medicine, Seoul, Republic of Korea

**Keywords:** Gastric cancer, Disability-adjusted life years (DALYs), Years of life lost (YLL), Years lived with disability (YLD), Global burden of disease (GBD), Korean national burden of disease (KNBD), Survivorship, Health policy, Korea, Japan

## Abstract

**Background:**

Global Burden of Disease (GBD) estimates are widely used for international health comparisons, but their validity in high-data settings remains debated. Gastric cancer provides a critical case, given its high incidence in East Asia and the availability of robust national screening and mortality data.

**Methods:**

We compared estimates from the Korean National Burden of Disease (KNBD) study and the GBD for gastric cancer between 2008 and 2018. We additionally examined Japan, another country with nationwide gastric cancer screening and high-quality mortality reporting, to assess whether similar patterns emerged.

**Results:**

KNBD reported declines in years of life lost (YLLs), reflected in the decreasing YLL share of disability-adjusted life years (DALYs), alongside substantial increases in years lived with disability (YLDs), underscoring survivorship-related disability. By contrast, GBD Korea showed a decrease in YLL but virtually no change in YLD. In Japan, where mortality has also declined substantially through national screening programs, GBD nevertheless reported negligible changes in YLL and YLD shares of total DALYs. Although a Japanese national DALY study is not available for direct comparison, these similar patterns across GBD Korea and GBD Japan raise concerns about the capacity of GBD methods to adequately capture survivorship in high-data countries.

**Conclusion:**

Our findings demonstrate that the same disease can generate fundamentally different burden-of-disease narratives depending on the metric framework applied. In Korea, national data highlight survivorship-related disability that is effectively absent in GBD estimates; in Japan, GBD may similarly downplays disability despite declining mortality. Policymakers may consider national burden-of-disease estimates as more appropriate for local planning, while global models could be strengthened by integrating high-quality country-level data to better reflect survivors’ burdens.

**Supplementary Information:**

The online version contains supplementary material available at 10.1186/s13690-025-01801-2.

**Table:** 

Text box 1. Contributions to the literature
• This study demonstrates that divergent DALY frameworks produce contrasting portrayals of gastric cancer burden in Korea: KNBD emphasizes survivorship-related disability, whereas GBD prioritizes mortality.
• This study identifies structural differences in mortality inputs, life-table assumptions, and disability modeling as the primary drivers of these discrepancies, rather than random variation.
• The findings underscore the value of national burden-of-disease studies for policy planning and call for global models to more explicitly integrate locally derived epidemiologic data.

## Background

Summary measures of population health, such as disability-adjusted life years (DALYs), were designed to provide standardized benchmarks across diseases, populations, and time. Since its inception, the Global Burden of Disease (GBD) project has established DALYs as the lingua franca of health priority-setting worldwide [1]. Yet GBD is not the only system in use. Countries with strong epidemiologic infrastructure, including Korea, have developed national burden of disease studies that draw directly on registry-based and survey data [2, 3].

Gastric cancer illustrates the stakes of these methodological differences. Although its global incidence has declined, it remains a leading cause of cancer burden, particularly in East Asia [4]. Korea and Japan, two of the world’s highest-incidence countries, have experienced profound population-level consequences from gastric cancer [5]. Over the past two decades, both nations have implemented nationwide screening programs that have substantially reduced mortality, reshaping the disease’s epidemiology [6, 7]. In Korea, the Korean National Burden of Disease (KNBD) study derives estimates from national registries, vital statistics, and locally specific life Tables (2, 3). By contrast, the GBD study, led by the Institute for Health Metrics and Evaluation (IHME), applies standardized global modeling methods, ensemble mortality estimation, and universal reference life Tables (8, 9).

Despite their shared aim of quantifying health loss, KNBD and GBD generate strikingly different estimates of gastric cancer burden in Korea. These divergences are not merely technical—they alter how health systems allocate resources, which priorities are emphasized, and which patient experiences are rendered visible [10]. Japan, although lacking a parallel national burden study, has followed a similar trajectory of mortality reduction through screening, further underscoring the importance of aligning global models with local epidemiologic realities [11].

Here, we systematically compare KNBD and GBD estimates of gastric cancer burden in Korea from 2008 to 2018. Our goal is not only to document these discrepancies but also to trace their methodological origins and examine their policy implications. In doing so, we highlight the need to integrate national epidemiologic data into global models and contribute to broader debates on how burden metrics capture—or obscure—the realities of high-survival cancers.

## Methods

### Data sources

We obtained gastric cancer burden estimates from the Korean National Burden of Disease (KNBD) study and its supplementary publications [2, 3, 12], as well as from the publicly available Global Burden of Disease (GBD) results tool maintained by the Institute for Health Metrics and Evaluation (IHME) through the Global Health Data Exchange (GHDx) [13] (Table 1). These estimates were drawn from existing national and global burden frameworks rather than recalculated from raw datasets.” We adhered to the Guidelines for Accurate and Transparent Health Estimates Reporting (GATHER) standards (see Additional file 1). This study also followed the STROBE (Strengthening the Reporting of Observational Studies in Epidemiology) guideline for observational studies [14].

**Table 1:** Comparison of data sources and analytic methods between the global burden of disease and Korean National burden of disease studies

Domain	GBD	KNBD
Data Sources	Utilizes a wide range of sources, including international and national surveys (e.g., Korea National Health and Nutrition Examination Survey).	Relies primarily on domestic data sources, especially National Health Insurance Service (NHIS) claims data.
YLD Calculation	Prevalence-based approach: prevalence × disability weight	Incidence-based approach: incidence × average duration of disability × disability weight
Disability Weights	Derived from international community surveys and applied uniformly across all locations and years. DWs were not stratified by cancer type.	A country-specific system of disability weights derived from medical professionals to better reflect Korean social preferences. DWs stratified by cancer type.
Stratification	National-level (no sub-regional estimates within Korea) Includes age stratification and GBD standard age-standardization.	Provides estimates for 250 sub-regions and incorporates socioeconomic stratification using income quintiles

Legend: *GBD* Global Burden of Disease, *KNBD* Korean National Burden of Disease, *YLDs* years lived with disability, Disability Weights * DWs*

The two systems draw on distinct data infrastructures. The KNBD relies primarily on domestic data sources, with a particular emphasis on National Health Insurance Service (NHIS) claims data [15]. Given Korea’s universal health coverage, NHIS captures virtually all insured medical service utilization, making it one of the most representative and comprehensive health databases worldwide [2]. In contrast, the GBD aggregates a wide range of international and national sources, including household and health surveys such as the Korea National Health and Nutrition Examination Survey (KNHANES) [13]. While GBD may include NHIS data for some models, it does not systematically utilize it, which introduces differences in representativeness and granularity between the two systems [16].

### Burden measures

Both the KNBD and GBD rely on the three standard measures of disease burden: Years of Life Lost (YLL), Years Lived with Disability (YLD), and Disability-Adjusted Life Years (DALY). Despite this shared framework, their methodologies diverge in important ways.

For YLL, the KNBD calculated values from observed gastric cancer deaths captured in the national vital registration system by year, age, and sex, then multiplying these counts by the remaining life expectancy at the age of death as derived from locally constructed life Table [17]. Life tables were constructed by Statistics Korea. The number of deaths and total population were adjusted then used to calculate mortality rates, which were then converted into death probabilities using a Brass-logit model and further adjusted using Greville’s 9th-degree smoothing coefficients. For ages 85 and older, death probabilities are estimated using the Coale-Kisker model. Death probabilities were then converted into life expectancies. By contrast, the GBD employed ensemble-modeled cause-age-sex-location-year-specific mortality estimates and applied a universal reference life table reflecting the global life expectancy [9]. The GBD 2021 cause-of-death database includes multiple data types capture the widest array of information, processed, corrected, and standardized so that they can be compared by cause, age, sex, location, and time. All-cause mortality is aggregated then redistributed across cause, age, sex, location, and time [9].

The approaches to YLD are also distinct. KNBD used an incidence-based method, computing YLD as the product of newly diagnosed gastric cancer cases, the expected average disease duration, and disability weights [15]. Expected disease duration was calculated using a DisMod-II program. Disability weights (DWs) were derived from Korean survey-based valuation studies derived from medical professionals, designed to reflect social preferences specific to the Korean population [12]. The GBD, in contrast, used a prevalence-based approach, multiplying sequela-specific prevalence by globally standardized disability weights and adjusting for comorbidity [8]. Disease-specific prevalence was modelled using DisMod-MR 2.1, then split into sequela-specific prevalence across a spectrum of severity. Depending on the availability of data, GBD either directly measured prevalence or extrapolated it from incidence and mortality.

A critical methodological distinction lies in the construction of disability weights (DWs). The KNBD developed its own country-specific system, ranging from 0 (full health) to 1 (death), and updated these weights in 2020 to capture the impact of the COVID-19 pandemic on disease burden [1]. Importantly, KNBD calculated DWs by cause and, for some conditions, subdivided them further by severity; for example, stomach cancer was classified into stages 1 through 4. The GBD, on the other hand, applied disability weights derived from international community surveys uniformly across countries and years, distinguishing only by severity level and without cause-specific differentiation [6].

In summary, KNBD applies a national framework rooted in medical professional input, producing cause-specific and severity-specific weights, while GBD employs a global framework based on community surveys that is limited to severity-specific distinctions.

### Disability-adjusted life years (DALYs)

In both systems, DALYs were defined as the sum of YLLs and YLDs. However, the methodological divergences in mortality estimation, disability weight assignment, and incidence versus prevalence modeling led to substantial differences in the portrayal of gastric cancer burden.

### Stratification

Another major difference between KNBD and GBD lies in stratification. The GBD generates only national-level estimates, applying age stratification along with its standard age-standardization protocol. For our analyses, we relied on the all-age estimates from the GBD to maintain consistency with KNBD outputs. By contrast, KNBD provides much finer resolution, producing estimates for 250 sub-regions across Korea and incorporating socioeconomic stratification by income quintiles, using insurance premiums as a proxy for household income. Mortality estimates in KNBD are further stratified by applying income-specific death distributions from Statistics Korea, and for injuries, prevalence and incidence distributions are drawn from the Korea Community Health Survey (KCHS). Notably, KNBD does not apply age standardization, which further differentiates its estimates from GBD outputs.

### Analytic framework

We selected 2008 and 2018 as reference years to capture a decade marked by substantial reductions in gastric cancer mortality in Korea following the expansion of national screening programs. Analyses assessed absolute estimates of YLL, YLD, and DALYs; their proportional changes over time; and the relative contributions of YLL and YLD, expressed as their ratios to total DALYs.

### Cross-country comparison

Finally, to contextualize findings, we retrieved GBD-based estimates for Japan, another high-incidence country with a national gastric cancer screening program. Unlike Korea, Japan does not maintain a parallel National Burden of Disease system; thus, only GBD data were available for cross-country comparison. In addition, GBD estimates for the United States were included as a benchmark representing a low-incidence country without a national screening program, to illustrate that the static proportion of YLD in GBD is not unique to East Asia but reflects a consistent feature of its global model.

## Results

Analyses encompassed the entire Korean population (~ 50 million) captured in the KNBD and national-level modeled estimates from the GBD, with uncertainty intervals provided in Table 2.

**Table 2:** Gastric cancer burden estimates from the Korean National burden of disease and global burden of disease studies for Korea, Japan, and the united States, 2008–2018 (All-Age rate and percentage Change)

	KNBD			GBD Korea			GBD Japan			GBD US		
	2008	2018	Change (%)	2008	2018	Change (%)	2008	2018	Change (%)	2008	2018	Change (%)
DALYs	333	289	−13.3	627 [549–733]	487 [418–625]	−22.3	865 [799–903]	735 [657–779]	−15.0	114 [108–117]	109 [103–113]	−4.4
YLLs	200	131	−34.4	611 [535–715]	474 [406–608]	−22.4	840 [775–876]	713 [636–754]	−15.1	111 [105–114]	106 [100–110]	−4.5
Proportion of YLLs (%)	60.1	45.3	−24.6	97.4	97.3	−0.1	97.1	97.0	−0.1	97.4	97.2	−0.2
YLDs	134	158	18.2	15 [11–21]	14 [9–19]	−6.7	25 [19–32]	23 [17–29]	−8.0	3 [2–3]	3 [2–3]	0.0
Proportion of YLDs (%)	40.2	54.7	36.1	2.4	2.9	20.8	2.9	3.1	6.9	2.6	2.8	7.7

Legend: *DALYs* disability-adjusted life years, *YLLs* years of life lost, *YLDs* years lived with disability, *KNBD* Korean National Burden of Disease, *GBD* Global Burden of Disease

Values are presented as all-age rates per 100,000 population

Percentage change refers to relative change between 2008 and 2018

### Disability-adjusted life years (DALYs)

Between 2008 and 2018, KNBD DALYs decreased from 333 to 289 (**−** 13.3%), reflecting simultaneous declines in YLL and increases in YLD share. By comparison, GBD–Korea DALYs also declined (627 [549–733] → 487 [418–625], **−** 22.3%), yet the composition remained dominated by YLL (> 97% throughout). GBD–Japan showed modest DALY reductions (865 [799–903] → 735 [657–779], **−** 15.0%), with similarly stable YLL and YLD shares above 97% and less than 3%, respectively. Thus, KNBD depicted a shifting burden marked by a rising contribution of YLD, whereas GBD estimates across all settings portrayed DALYs as almost entirely driven by YLL.

### Years of life lost (YLL)

Between 2008 and 2018, KNBD YLL fell from 200 to 131 (− 34.4%), and the YLL share of DALYs declined from 60.1% to 45.3% (− 24.6%). By contrast, GBD–Korea YLL fell from 611 [535–715] to 474 [406–608] (− 22.4%), yet YLL continued to account for ~ 97% of DALYs (97.4% → 97.3%, − 0.1%). In 2018, GBD–Korea YLL remained about 3.6× higher than KNBD (474 vs. 131). A similar pattern appears in GBD–Japan (YLL 840 [775–876] → 713 [636–754], **−** 15.1%; YLL share 97.1% → 97.0%, **−** 0.1%).

### Years lived with disability (YLD)

From 2008 to 2018, KNBD YLD increased from 134 to 158 (+ 18.2%), with its proportional share of DALYs rising from 40.2% to 54.7% (+ 36.1%). In contrast, GBD–Korea YLD declined slightly (15 [11–21] → 14 [9–19], **−** 6.7%), with a negligible change in its proportional share. A similar pattern was observed in GBD–Japan (YLD 25 [19–31] → 23 [17–29], **−** 8.0%; share 2.9% → 3.1%). Thus, in 2018, KNBD attributed over half of the gastric cancer burden in Korea to YLD, whereas GBD estimates for all countries kept YLD below 3% of DALYs.

### Korea–Japan comparison

In Japan, where national burden-of-disease data are not available, only GBD estimates were available for comparison. These showed patterns similar to those observed in Korea’s GBD estimates: approximately 97% of gastric cancer DALYs were attributed to YLL, with less than 3% to YLD, and no major shifts between 2008 and 2018. Thus, while Korea’s national data revealed large declines in YLL and substantial YLD contributions, Japan’s GBD-only profile resembled Korea’s GBD estimates, with DALYs almost entirely composed of YLL.

## Discussion

The comparison between KNBD and GBD estimates of gastric cancer burden in Korea reveals not merely technical discrepancies but differing analytic frameworks and purposes. KNBD characterizes gastric cancer as a complex and evolving public health challenge—one increasingly shaped by survivorship, post-treatment sequelae, and healthcare utilization in addition to mortality. In contrast, GBD’s estimates, designed for international comparability, rely primarily on mortality-based modeling and standardized life table assumptions, which can underrepresent the contribution of post-diagnosis survival and chronic morbidity to total disease burden in high-survival settings. This divergence reflects differing aims: GBD provides a harmonized global benchmark, whereas national frameworks such as KNBD are better suited to capture the lived epidemiology and healthcare realities that inform domestic cancer control priorities.

### Discrepancies in years of life lost (YLL)

The most immediate puzzle lies in YLL trends. National vital statistics and cancer registry data from Korea document a substantial decline in gastric cancer mortality between 2008 and 2018, largely attributed to the expansion of the national endoscopic screening program, widespread *Helicobacter pylori* eradication efforts, and improvements in surgical and systemic therapy outcomes [18, 19]. In relative terms, this corresponds to roughly a one-third decline in age-standardized gastric cancer mortality, consistent with national vital-statistics trends during the same period [2].GBD’s YLL estimates show a more modest reduction, with values remaining several times higher than those derived from KNBD. This disconnect is not a matter of minor calibration but of fundamentally different approaches to mortality modeling and life table assignment.

KNBD applies national mortality data, linked directly to Statistics Korea life tables. This provides an internally consistent framework: when mortality decreases by 40%, YLL falls proportionally, because life expectancy at each age is fixed by the national standard. By contrast, GBD employs the Cause of Death Ensemble model (CODEm), redistributes “garbage codes,” and then applies a universal reference life table that is not directly available for verification. By applying a covariate selection algorithm across four model classes including mixed-effects, and spatial-temporal Gaussian Process Regression (ST-GPR) [20], CODEm aggregates multiple predictors into an ensemble optimized for out-of-sample-predictive performance [21]. These methodological layers are designed to ensure cross-country comparability, but they appear to dampen real mortality improvements, particularly in countries where rapid progress has occurred through population screening, improved treatment, and risk factor reduction. While the CODEm framework recommends that for GBD studies, estimates should be generated with the most high-quality data available in order to capture spatial-temporal patterns, the observed patterns in gastric cancer YLL trends for Korea suggest that high-quality national mortality data may have been underrepresented in the modeling process [22]. The implication is more than technical. If global metrics understate mortality declines, they risk obscuring the success of national programs. In the case of Korea, decades of investment in population-based endoscopic screening and H. pylori eradication have achieved measurable reductions in gastric cancer mortality, with the Korean National Cancer Screening Program for gastric cancer believed to have reduced cause-specific mortality by 21–33% since 2002 [18, 19]. Yet GBD portrays Korea as a country with persistently high and slow-to-decline YLL. For policymakers, this could weaken support for screening or prevention, since the burden appears resistant to intervention.

### The invisibility of years lived with disability (YLD)

If YLL tells a story of under-recognized progress, YLD reveals lower rates of survivorship burden from GBD estimates. KNBD attributes approximately 45–55% of total DALYs to YLD, reflecting the long-term health consequences of gastric cancer treatment. By contrast, GBD estimates YLDs by calculating cause-age-sex-location-year-specific prevalence of sequelae and multiplying the prevalence rates for each disease by varying disability weights [23]. While this method allows for the adjustment of comorbidities [24], only 2–3% of DALYs are assigned to YLD, reducing gastric cancer to a disease of premature death with limited recognition of survivorship-related disability [25].

This gap reflects both methodological and cultural choices. KNBD defines YLD as incidence × duration × disability weight, implicitly capturing the multi-year sequelae that follow diagnosis and treatment. Because this incidence-based approach recalculates YLDs each year as new cohorts of survivors enter the system, it is inherently more sensitive to changes in survivorship patterns—particularly in countries like Korea, where early detection and long-term survival are increasing [26]. Although incidence and duration inputs are not always transparent, the underlying assumption is that survivors live with substantial impairments for extended periods.

In contrast, the GBD’s prevalence-based approach offers a more cross-sectional snapshot of disability at a single point in time. While this can produce a stable estimate across populations, it may underrepresent the evolving burden of chronic sequelae among new survivor cohorts, and has been noted by scholars as a limitation for studies where accumulated comorbidities is a common phenomenon [27]. Consequently, GBD’s approach captures prevalence of disability rather than its dynamic accumulation—potentially missing shifts in survivorship epidemiology that incidence-based systems like KNBD are better equipped to detect.

This limitation is particularly evident in GBD 2021, where the sequelae for cancer are restricted to broad phases—diagnosis and primary therapy, remission, disseminated/metastatic, and terminal—without explicit representation of long-term survivorship condition [28]. Because of this structural simplification, the model focuses largely on the acute phases of illness, overlooking the extended survivorship period that increasingly defines the gastric cancer experience in high-survival settings. The set of sequelae included is therefore narrow, often confined to acute treatment phases or generic categories such as moderate abdominal pain. As a result, post-gastrectomy syndromes—dumping, malnutrition, anemia, reflux, and chronic fatigue—are rarely represented, despite being common and clinically significant in long-term survivors [8]. This omission helps explain why the GBD framework underestimates the cumulative disability burden that Korean and Japanese clinicians routinely observe in gastric cancer survivors.

The cultural component is critical. In the GBD framework, disability weights are derived from general population judgments about health states, not from patients or clinicians living with the conditions. In many Western contexts, where gastric cancer is rare and survivorship less common, the daily struggles of living without a stomach are poorly recognized. By contrast, in Korea and Japan, where survival is more common and post-treatment sequelae are part of collective medical experience, survivorship burden is well understood. Reflecting this, the KNBD instead uses disability weights derived from medical professionals, allowing for cause-specific and severity-specific valuation that more directly captures the realities of survivorship. The relative underweighting of YLD in GBD estimates may reflect underlying assumptions about how disability and survivorship are valued in global health metrics.

### Broader methodological and cultural implications

These discrepancies underscore a fundamental tension in global burden estimation. GBD prioritizes comparability: by applying standardized inputs, disability weights, and reference life tables, it enables uniform metrics across countries. This commitment to comparability of measurement, allows for decision-makers to interpret differences in disease burden trends within a unified, global framework that ensures measures carry the same meaning regardless of place or period [29]. It is also noteworthy that because of this commitment to international comparability, the GBD recomputes entire historical time series datasets for a disease, injury or risk factor when changes in case definitions do not lead to spurious comparisons with past assessments [29]. Yet this very comparability comes at the cost of validity in settings where national data and lived experiences diverge from global assumptions.

From a methodological perspective, the divergence highlights the need to incorporate high-quality national datasets more directly into global models. Korea and Japan maintain robust cancer registries, claims data, and life tables that can provide more accurate inputs than modeled estimates. By sidelining these sources, GBD risks producing metrics that are globally comparable but locally implausible.

From a cultural perspective, the undervaluation of YLD raises ethical concerns. What counts as “disability” is not a purely biological fact but a socially mediated judgment. If those judgments are derived primarily from populations unfamiliar with gastric cancer survivorship, they risk erasing the burdens of millions of patients worldwide. This is not limited to gastric cancer. Other high-survival cancers—such as breast and colorectal cancer—may also suffer from underestimated YLD in GBD, distorting resource allocation away from survivorship care [30].

### Policy implications of measurement bias

How disease burden is quantified has direct consequences for policy. When YLL is overemphasized, interventions focus narrowly on mortality reduction, obscuring the chronic morbidity many survivors face. Even after curative surgery, patients often live with lasting nutritional, functional, and psychosocial impairments—burdens better captured by YLD but often underrepresented when global metrics minimize disability.

In countries such as Korea and Japan, where national screening has already driven down gastric cancer mortality, the emerging priority is not simply to prevent premature death but to reduce the chronic disability that accompanies survivorship. If YLD is underestimated, health systems will continue to overinvest in mortality reduction and underinvest in survivorship care—a paradox in which those most in need of disability-focused interventions are least visible in burden estimates.

Primary prevention offers the most powerful pathway to reducing both YLD and YLL by preventing incident disease through H. pylori eradication, risk factor modification, and population-level strategies. Yet primary prevention must be complemented by secondary prevention—that is, the early detection of premalignant or early-stage disease through screening and timely treatment. Together, these approaches not only extend life but also reduce the disability that can persist after survival.

The lessons from Korea and Japan are therefore instructive. In Japan, where only GBD estimates are available, the same YLL-dominant pattern emerges, underscoring how global methods can obscure the visibility of survivorship burden in high-incidence countries. Effective cancer control thus requires a dual strategy: mortality reduction through secondary prevention remains vital, but it must be matched by investment in primary prevention to lower incidence and address the survivorship burden that follows. Recognizing and addressing the full spectrum of disease burden—mortality and morbidity—will be essential to guide balanced, forward-looking investments in cancer prevention, treatment, and survivorship care.

### Limitations

This study has several limitations that should be acknowledged. First, our comparison relies on secondary estimates produced by KNBD and GBD, rather than on direct re-estimation from raw data. As such, we are constrained by the assumptions, coding decisions, and data availability within each framework. Second, for Japan we lacked a parallel national burden-of-disease system, which limited our ability to directly assess whether KNBD-like patterns would emerge in another high-incidence setting; instead, our conclusions rest on indirect comparison using GBD estimates alone. Third, although we highlight survivorship-related disability as a key divergence, our analysis does not disentangle the relative contributions of incidence, duration, and disability weight assumptions within each system. Fourth, we implicitly assume that declines in mortality translate into proportional reductions in YLL; however, YLL is also influenced by the age at which deaths occur. For example, deaths occurring at younger ages contribute disproportionately higher YLL due to longer expected life remaining, while deaths at older ages contribute less. Thus, even as mortality decreases, the relative share of YLL may not decline in a strictly proportional fashion. Fifth, GBD estimates rely heavily on complex modeling processes such as CODEm and standardized disability weights, with limited transparency around how country-level inputs are weighted or adjusted; these differences may themselves drive some of the observed discrepancies. Finally, while we focus on gastric cancer, patterns may not generalize to other cancers or chronic conditions where survivorship plays a different role. Despite these caveats, the consistency of discrepancies observed across metrics, time points, and countries suggests that our findings reflect structural features of the DALY frameworks rather than random variation.

## Conclusions

Differences between KNBD and GBD in estimating gastric cancer burden reflect variation in data sources, methodological assumptions, and cultural framing. Whereas GBD places near-exclusive weight on mortality, KNBD highlights the shifting balance between fatal and non-fatal components, capturing both the decline in YLL and the rising prominence of YLD. In doing so, KNBD portrays gastric cancer as a condition in transition—from one defined primarily by premature death to one increasingly shaped by survivorship. GBD, in contrast, continues to depict it as overwhelmingly fatal. These perspectives are divergent and sometimes conflicting, yet considered together, they provide a more comprehensive picture of disease burden.

To strengthen policy relevance, global burden assessments should more fully incorporate high-quality national registries and survivorship data. While this integration is technically feasible, it would require collaborative data-sharing mechanisms and alignment with existing GBD modeling processes. At the same time, reducing the future burden requires equal attention to primary prevention, particularly interventions that lower incidence through early detection and eradication of key risk factors. Korea’s experience illustrates how local insights can refine global metrics, ensuring that health policies address not only the imperative of reducing mortality but also the need to prevent disease and support long-term survivorship.

## Supplementary Information

Supplementary Material 1

## Data Availability

The datasets analyzed during the current study are available via the online GBD results tool (https://vizhub.healthdata.org/gbd-results/) and KNBD studies (2, 3).

## Abbreviations

GBD: Global Burden of Disease
KNBD: Korean National Burden of Disease
DALYs: Disability-adjusted life years
YLLs: Years of life lost
YLDs: Years lived with disability
IHME: Institute for Health Metrics and Evaluation
GHDx: Global Health Data Exchange
GATHER: Guidelines for Accurate and Transparent Health Estimates Reporting
NHIS: National Health Insurance Service
KNHANES: Korea National Health and Nutrition Examination Survey
DWs: Disability weights
KCHS: Korea Community Health Survey
CODEm: Cause of Death Ensemble model
ST-GPR: Spatial-temporal Gaussian Process Regression
