# Supplementary material for: One cancer, two stories: divergent estimates of gastric cancer burden in Korea
Source: Arch Public Health. 2026 Jan 14;84:13. doi: 10.1186/s13690-025-01801-2 (PMC12801540; doi:10.1186/s13690-025-01801-2)
Supplement: Supplementary file 1 — Supplementary Material 1 [file 13690_2025_1801_MOESM1_ESM.docx]

***Additional File 1:***

***Supplementary material for One Cancer, Two Stories: Divergent Estimates of Gastric Cancer Burden in Korea***

## Supplemental table 1: GATHER checklist

| # | Checklist item | Section/paragraph/ interpretation |
| --- | --- | --- |
| Objectives and funding | | |
| 1 | Define the indicators, populations, and time periods for which estimates were made. | Methods / “Data Sources”, “Burden Measures”, “Stratification” and “Analytic Framework” sections |
| 2 | List the funding sources for the work. | No funding |
| Data inputs | | |
| *For all data inputs from multiple sources that are synthesized as part of the study:* | | |
| 3 | Describe how the data were identified and how the data were accessed. | Methods / “Data Sources” |
| 4 | Specify the inclusion and exclusion criteria. Identify all ad-hoc exclusions. | Methods / “Data Sources”, “Stratification” and “Analytic Framework” |
| 5 | Provide information on all included data sources and their main characteristics. For each data source used, report reference information or contact name/institution, population represented, data collection method, year(s) of data collection, sex and age range, diagnostic criteria or measurement method, and sample size, as relevant. | Methods / “Data Sources”, “Stratification” and “Analytic Framework”. Data available via online data source tools (<http://ghdx.healthdata.org/gbd-2021/data-input-sources>) and KNBD studies [1, 2]. |
| 6 | Identify and describe any categories of input data that have potentially important biases (e.g., based on characteristics listed in item 5). | Methods / “Data Sources”, “Burden Measures”, “Stratification” and “Analytic Framework” sections |
| *For data inputs that contribute to the analysis but were not synthesized as part of the study:* | | |
| 7 | Describe and give sources for any other data inputs. | Data available via online data source tools (<http://ghdx.healthdata.org/gbd-2021/data-input-sources>) and KNBD studies [1, 2]. |
| *For all data inputs:* | | |
| 8 | Provide all data inputs in a file format from which data can be efficiently extracted (e.g., a spreadsheet as opposed to a PDF), including all relevant meta-data listed in item 5. For any data inputs that cannot be shared due to ethical or legal reasons, such as third-party ownership, provide a contact name or the name of the institution that retains the right to the data. | Data available via online data source tools (<http://ghdx.healthdata.org/gbd-2021/data-input-sources>) and KNBD studies [1, 2]. |
| Data analysis | | |
| 9 | Provide a conceptual overview of the data analysis method. A diagram may be helpful. | Details on overall methodological processes have been published previously [1-4]. |
| 10 | Provide a detailed description of all steps of the analysis, including mathematical formulae. This description should cover, as relevant, data cleaning, data pre-processing, data adjustments and weighting of data sources, and mathematical or statistical model(s). | Methods / “Data Sources”, “Burden Measures”, “Stratification” and “Analytic Framework” sections. Details on methodological processes have been published previously [1-4]. |
| 11 | Describe how candidate models were evaluated and how the final model(s) were selected. | Methods / “Burden Measures” section. Details on methodological processes have been published previously [1-4]. |
| 12 | Provide the results of an evaluation of model performance, if done, as well as the results of any relevant sensitivity analysis. | Methods / “Burden Measures” section. Details on methodological processes have been published previously [1-4]. |
| 13 | Describe methods for calculating uncertainty of the estimates. State which sources of uncertainty were, and were not, accounted for in the uncertainty analysis. | Details on methodological processes have been published previously [1-4]. |
| 14 | State how analytic or statistical source code used to generate estimates can be accessed. | Details on methodological processes have been published previously [1-4]. |
| Results and discussion | | |
| 15 | Provide published estimates in a file format from which data can be efficiently extracted. | Data available via online data results tools (<https://vizhub.healthdata.org/gbd-results>) and KNBD studies [1, 2]. |
| 16 | Report a quantitative measure of the uncertainty of the estimates (e.g. uncertainty intervals). | Data available via online data results tools (<https://vizhub.healthdata.org/gbd-results>) and KNBD studies [1, 2]. |
| 17 | Interpret results in light of existing evidence. If updating a previous set of estimates, describe the reasons for changes in estimates. | Discussion |
| 18 | Discuss limitations of the estimates. Include a discussion of any modelling assumptions or data limitations that affect interpretation of the estimates. | Discussion |

**References:**

1. Jung Y-S, Kim Y-E, Ock M, Yoon S-J. Measuring the Burden of Disease in Korea Using Disability-Adjusted Life Years (2008–2020). jkms. 2024;39(7):e67–0.

2. Jung Y-S, Yoon S-J. Trends and Patterns of Cancer Burdens by Region and Income Level in Korea: A National Representative Big Data Analysis. crt. 2022;55(2):408–18.

3. Ferrari AJ, Santomauro DF, Aali A, Abate YH, Abbafati C, Abbastabar H, et al. Global incidence, prevalence, years lived with disability (YLDs), disability-adjusted life-years (DALYs), and healthy life expectancy (HALE) for 371 diseases and injuries in 204 countries and territories and 811 subnational locations, 1990&#x2013;2021: a systematic analysis for the Global Burden of Disease Study 2021. The Lancet. 2024;403(10440):2133–61.

4. Naghavi M, Ong KL, Aali A, Ababneh HS, Abate YH, Abbafati C, et al. Global burden of 288 causes of death and life expectancy decomposition in 204 countries and territories and 811 subnational locations, 1990&#x2013;2021: a systematic analysis for the Global Burden of Disease Study 2021. The Lancet. 2024;403(10440):2100–32.
